# Current perspectives on clinical use of exosomes as novel biomarkers for cancer diagnosis

**DOI:** 10.3389/fonc.2022.966981

**Published:** 2022-08-31

**Authors:** Xiaomei Yi, Jie Chen, Defa Huang, Shuo Feng, Tong Yang, Zhengzhe Li, Xiaoxing Wang, Minghong Zhao, Jiyang Wu, Tianyu Zhong

**Affiliations:** ^1^ The First School of Clinical Medicine, Gannan Medical University, Ganzhou, China; ^2^ Laboratory Medicine, First Affiliated Hospital of Gannan Medical University, Ganzhou, China; ^3^ English Teaching and Research Section, Gannan Healthcare Vocational College, Ganzhou, China

**Keywords:** exosomes, extracellular vesicles (EVs), biogenesis, exogenous factors, release, molecular cargoes, diagnostics, biomarkers

## Abstract

Exosomes are a heterogeneous subset of extracellular vesicles (EVs) that biogenesis from endosomes. Besides, exosomes contain a variety of molecular cargoes including proteins, lipids and nucleic acids, which play a key role in the mechanism of exosome formation. Meanwhile, exosomes are involved with physiological and pathological conditions. The molecular profile of exosomes reflects the type and pathophysiological status of the originating cells so could potentially be exploited for diagnostic of cancer. This review aims to describe important molecular cargoes involved in exosome biogenesis. In addition, we highlight exogenous factors, especially autophagy, hypoxia and pharmacology, that regulate the release of exosomes and their corresponding cargoes. Particularly, we also emphasize exosome molecular cargoes as potential biomarkers in liquid biopsy for diagnosis of cancer.

## Introduction

Extracellular vesicles (EVs) are secreted from almost all cell types (1), and widely distributed in various body fluids, such as urine (2), blood (3), milk (4), saliva (5), cerebrospinal fluid (6), amniotic fluid (7) and semen (8), can transmit information between cells and participate in many physiological and pathological processes. It is known that the extraction and isolation of exosomes from different body fluids are mainly achieved by ultracentrifugation, ultrafiltration, sedimentation, density gradient centrifugation, immune-capture, precipitation and commercial reagents(**Table 1**). Exosomes are bi-layered lipid vesicles produced by the endosomal pathway, a subset of EVs with a diameter of 30-150nm (36, 37). However, due to the limitations of the isolation method, we usually define the particles less than 200nm in diameter are exosomes. Therefore, the International Society of Extracellular Vesicle (ISEV) statement in the Minimum Information on Extracellular Vesicle Research 2018 (MISEV2018) recommends the use of “EVs” as a general term (36). In this review, EVs mainly refer to exosomes without special instructions.

**Table 1:** Methods for isolation of exosomes from different biological sample types.

Sample types	Isolation methods	Types of cargo	References
Urine	UC,UF,DGC,SEC,PC,PEG,IC,MF,CRG	Proteins, MiRNAs, Lipids	(9–12)
Blood	UC,UF,DGC,SEC,PC,PEG,IC,MF,CRG	Proteins, MiRNAs, Lipids	(13–17)
Milk	UC,UF, DGC,SEC,PC,CRG	Proteins,RNAs,MiRNAs,Lipids	(18–21)
Saliva	UC, UF, DGC,SEC,PC,CRG	Proteins, MiRNAs	(22–25)
Cerebrospinal fluid	UC,UF,SEC, PC,CRG	Proteins, MiRNAs	(26–30)
Amniotic fluid	UC,UF,CRG	Proteins, MiRNAs,	(31, 32)
Semen	UC, UF,PC,PEG,CRG	Proteins, MiRNAs	(33–35)

UC, ultracentrifugation; UF, ultrafiltration; DGC, density gradient centrifugation; PC, precipitation PEG, polyethylene glycol precipitation; IC, immuno-capture; MF, microfluidics; SEC, size-exclusion chromatography; CRG, Commercial reagents.

**Figure 1:**
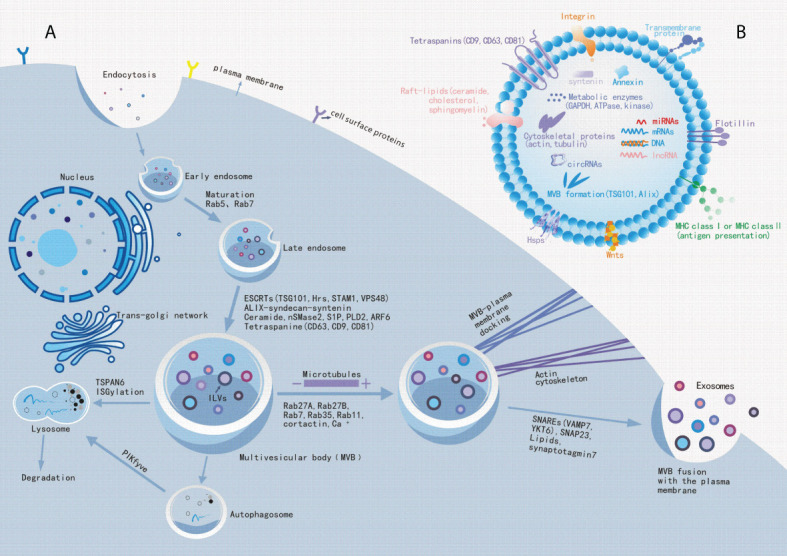
Exosome biogenesis. **(A)**: Schematic diagram of the molecular mechanisms of exosome biogenesis. Extracellular components, such as proteins, lipids, nucleic acids and small molecules, can enter cells with cell surface proteins through endocytosis and plasma membrane invagination. Under endocytosis, it leads to the formation of early endosomes and late endosomes, which bud out into multiple vesicles (MVBs) containing luminal vesicles (ILVs). Some molecules, such as ESCRT proteins (ALIX, TSG101, etc.), lipids and tetraspanin proteins mediate this process. Subsequently, MVBs will fuse to lysosomes or autophagosomes to accelerate their degradation to inhibit exosome release, or MVBs transported along the cytoskeleton and microtubule network to the plasma membrane after maturation, where it can fuse with the plasma membrane and release exosomes into extracellular space. Among these, Rabs, Actin and SNARE proteins are involved in exosome release.**(B)**: Exosome biomarkers. Exosomal luminal cargoes are mainly composed of proteins, lipids, nucleic acids, and other metabolites that can function in the recipient cells. Among these, CD9, CD63, CD81, flotillin, and Annexin can be used as exosome biomarkers.

Exosomes are present in biological fluids as a form of intercellular communication to transport proteins, lipids, nucleic acids, and metabolites to the pericellular environment (38, 39). Exosome biogenesis are tightly regulated, possibly by interactions with different effectors (40, 41), which mainly involved with ESCRT-dependent and ESCRT-independent mechanisms (42). Exosome biogenesis begins in the endocytic pathway, where the plasma membrane invagination packages cell membrane proteins and some extracellular components together to form the early endosomes (EEs) (43, 44). After that, EEs exchange substances with other organelles, or further mature into late endosomes (LEs), and the late endosomal membrane invaginate to form multiple vesicles (MVBs) containing luminal vesicles (ILVs). Next, MVBs bind to lysosomes or autophagosomes for degradation, or they are transported to the plasma membrane through the cytoskeleton and microtubule network, which then efflux to form exosomes (**Figure 1A**) (45–47). Interestingly, exosomal cargo molecules (**Figure 1B**) (proteins, lipids, and nucleic acids) regulate the whole process (42, 45, 48, 49). For example, tetraspanin proteins (e. g.: CD9, CD63, CD81, CD82), major histocompatibility complex (MHC) molecules, heat shock proteins (HSPs), endosomal sorting complex (ESCRT) proteins (e. g. Alix, TSG101), Rab proteins, actin, soluble N-acetamide sensitive factor attachment proteins (SNAREs) are the major participating proteins (50–53). Similarly, lipid components such as ceramide, cholesterol, phosphatidic acid, phosphatidylinositol 3-phosphate, phosphatidylinositol-3, 5-diphosphate, and sphingosine 1-phosphate are also involved in the process (54–57). A summary of the molecular cargoes associated with exosome biogenesis process is presented in **Table 2**.

**Table 2:** The Role of Related Molecular Cargoes in Exosome Formation.

Molecular Cargo Types	Process Involved	The Role Played in Exosome Formation	References
***Proteins***			
Tetraspanin proteins (e. g., CD9, CD63, CD81, CD82)	Exosome biogenesis, the targeting and release of exosomes cargo	Mediating the budding of ILVs and interacting with cholesterol to induce membrane curvature and the fusion of MVBs with the plasma membrane	(58–61)
Major histocompatibility composite (MHC) molecules (e. g., class MHC I and class MHC II)	Exosome biogenesis and antigen presentation	Mediating the budding of the ILVs	(62, 63)
Heat shock proteins (Hsps) (e. g. HSP90 and HSP70)	Exosome release and signaling	Induced membrane deformation and the fusion of MVBs with the plasma membrane	(64, 65)
ESCRT proteins (e. g., Alix, TSG101)	Exosome biogenesis	Interaction with the s yndecans-syntenin-Alix complex promotes the budding of ILVs	(66–68)
Rab proteins (e. g., Rab11, Rab35, Rab27A, and Rab27B)	Exosome biogenesis and release	Involved in vesicle budding, transport, and fusion	(69–71)
actin	Exosome release	Participating in the transport process of MVBs	(72, 73)
SNARE proteins	Exosome release	Induced fusion of MVBs with the plasma membrane	(74, 75)
***Lipids***			
ceramide	Exosome biogenesis and cargo sorting	Negative curvature of the induced membrane	(76, 77)
cholesterol	Exosome biogenesis, transport, and release	MVBs are induced to fuse with the plasma membrane, interact with ORP1L and control endosome movement along microtubules	(78–80)
sphingomyelin	Exosome biogenesis and signaling	Negative curvature of the induced membrane	(77, 81)
PA	Exosome biogenesis	Induced the negative curvature of the membrane, interacting with syntenin to recruit syndecan, CD63, and ALIX at the budding site	(82, 83)
Phosphatidylinositol 3-phosphate	Cargo sorting	Interaction with HRS proteins sorted cargo into endosomes and binding with ESCRT-0 in the membrane to recruit ESCRT-I, -II and-III	(84, 85)
Phosphatidylinositol-3, 5-diphosphate	Exosome release	Fusion with lysosomes regulates MVBs with lysosomal degradation	(86)
1-Sphingosine phosphate	Cargo sorting	Interactions with the inhibitory G protein-coupled S1P receptors in the MVBs membrane	(87)

Initial studies suggested that exosomes were some waste materials excreted by cells in order to maintain homeostasis (138). Recent reports suggest that exosomes are capable of material transport and information transfer between cells, thereby mediating many physiological and pathological processes (51, 72, 139, 140). Furthermore, these small vesicles are involved in immunomodulation and intercellular communication (141), and mediate the disease progression of cancer (142), cardiovascular disease (143–145), metabolic disease (146), degenerative change (147) and autoimmunity (148). It is currently believed that the key to exosomes biological functions lies in their molecular cargoes, including proteins, lipids, and nucleic acids. For example, phosphatidylinositol glycan-1 (GPC1) is a cell surface proteoglycan rich in cancer cell-derived exosomes, and Melo et al. (120) identified that GPC1 has the potential for early detection of pancreatic cancer lesions to promote the possibility of curative surgical treatment (120) found that CRC cell-derived exosomal HSPC111 protein promotes pre-metastatic niche formation and CRC liver metastases (CRLM) *via* reprogramming lipid metabolism in cancer-associated fibroblasts (CAFs), which implicate HSPC111 may be a potential therapeutic target for preventing CRLM (149). In addition, phosphatidylserine, cholesterol and ceramide are also play key roles in exosome formation, which affect cargo sorting, signaling and exosomes structure (150, 151). MicroRNAs(miRNAs) are one of the most abundant RNA species in exosomes, and miRNAs play roles in various biological processes such as exocytosis and exosome-mediated cellular communication (73, 152). For example, Fu et al. (153) found that exosomes content miR-98-5p inhibits the progression of pancreatic ductal adenocarcinoma(PDAC) by targeting MAPK signaling (153). In addition, microarray profiles identified that miR-106a-5p and miR-19b-3p were remarkably overexpressed in the serum exosomes of patients with gastric cancer(GC). Notably, integrating the two miRNAs could identify GC patients among healthy volunteers with a 0.814 area under the curve (AUC) value, which was higher than that obtained using CEA or AFP (154). *Of note*, the parental information of these exosomes may differ significantly between healthy people and patients, making some molecular cargoes in exosomes potentially as specific biomarkers of cancer. Importantly, the ability to selectively control the release of exosomes in pathological situations without compromising their role as essential components in physiological situations would make exosomes have promising clinical applications in disease diagnosis, treatment and prognosis. In this review, we conclude the role of exosomes molecular cargoes in their biogenesis. We also underline the potential mechanisms by which autophagy, hypoxia and pharmacology exogenous factors affect exosome release. And summarize the key roles of exosome molecular cargoes play in cancer diagnosis. Furthermore, we discuss the challenges and potential applications of exosomes research.

## Exogenous factors modulate exosome release

The biogenesis of exosomes is influenced by a variety of extrinsic factors in addition to the molecular correlation of the above-mentioned cargoes. A greater understanding of the underlying mechanisms that influence exosome release factors could provide new targets for disease diagnosis and treatment. The potential mechanisms by which autophagy, hypoxia, and pharmacological factors affect exosome release are presented below.

### Autophagy modulates exosome release

Autophagy is a process that causes the degradation of cellular material at the lysosome. Autophagosomes can fuse with MVBs or directly with lysosomes to degrade cargoes (155). It was found that autophagy-related proteins, such as ATG5 and ATG16L1, affects exosome release process. For example, Abdulrahma et al. reported that when the autophagy protein ATG5 was knocked down, it greatly promoted the release of prion protein (PRNP) exosomes (156). Recently, Zheng et al. demonstrated that sulforaphane inhibits autophagy and induces exosome release *via* regulating mTOR/TEF3 (157). In addition, Guo et al. showed that ATG16L1 and ATG5 autophagy proteins protected MVBs from lysosomal degradation and thus facilitated the fusion of MVBs with the plasma membrane to facilitate exosome release. Conversely, silencing of ATG16L1 and ATG5 decreased exosome release, probably due to the ability of ATG5 to separate ATP6V1E1 from V1V0-ATPase, thereby inhibiting MVBs acidification and facilitating exosome release (158). Crucially, Keller et al. identified that ATG proteins promoted exosome release through a lysosomal non-dependent pathway, i.e. secretory autophagy, which in turn excreted bacterial toxin receptors from the membrane surface in the form of exosomes, assisting host cells to resist toxin damage and enhancing the antimicrobial response of the organism (159). These studies all suggest that autophagy may play a specific role to affect exosome release.

### Hypoxia modulates exosome release

Hypoxia may affect exosome release through hypoxia-inducible factors (HIF), Rab-GTPases, NF-κB and four transmembrane protein signaling pathways, but the specific mechanisms involved remains unclear (160). Hypoxia-inducible factor (HIF) is a major component of the hypoxia-related signaling pathway that directly or indirectly regulates the process of exosome release. Recently, it has been reported that HIF mediates endocytosis mainly by increasing the expression of glucose transporter protein (GLUT-1), transferrin receptor and epidermal growth factor receptor (EGFR), which in turn induces exosome release (161). It was found that the increased release of exosomes from rat proximal renal tubular cells (RPTC) (162) and breast cancer cells (163) in hypoxic environment was mainly mediated by HIF-1α. In particular, hypoxia can cause glycolysis and lactate accumulation. Ban et al. demonstrated that exosome markers such as CD9, CD63, and HSP70 expression increased under acidic conditions and were more conducive to exosome release, whereas exosomal proteins and exosomal RNA were not detected in alkaline environments and exosome release was reduced (164). Wang et al. demonstrated that hypoxia increased the number of exosomes released from colorectal cancer cells compared to hyperoxic conditions (165). On the other hand, hypoxia not only alters exosome size, sorting mechanisms and exosome uptake and binding capacity in the tumor microenvironment, but also impacts exosome-mediated tumor biological functions (166). Interestingly, different hypoxic conditions, such as duration and severity of hypoxia, can have dramatically variable impacts on the amount and content of exosomes released by different cell types (167)

### Pharmacology modulates exosome release

Nowadays, utilizing exosome as nanomaterials for drug delivery is of great interest to researchers. Notably, drugs may have a dramatic impact on drug repositioning and as potential novel anticancer agents by affecting certain molecules in the exosome release process. However, there are no drugs available to control the production of harmful exosomes in tumor cells (168). PH and Ca2+ are required for exosome release. Amiloride is a drug that inhibits Na +/H + exchange pump and Na +/Ca 2+ channels, and Savina et al. demonstrated that it reduced exosome release (169). Importantly, amiloride inhibits ceramide formation by indirectly inhibiting acid sphingomyelinase (aSMase), which in turn inhibits exosome release (170). Similarly, promethazine, a tricyclic antidepressant, has been found to reduce exosome release through inhibition of aSMase activity in the prostate cancer cell line PC3 by Kosgodage et al. (171). Metformin is the first-line drug for the treatment of type 2 diabetes, which increases insulin sensitivity and reduces fat synthesis (172). Recently, Liao et al. have demonstrated that metformin promotes the fusion of MVBs with the plasma membrane through autophagy and thus increased exosome release from mesenchymal stem cells (MSCs), which improved their therapeutic effect on senescent cells (173). In addition, metformin may promote exosome release to regulate stress by increasing the production of reactive oxygen species in tumor cells (174). Gao al.demonstrated that all-trans retinoic acid suppressed GES-1 cell proliferation induced by exosomes from patients with precancerous lesions by arresting the cell cycle in S-phase (175). Therefore, these drugs may act by acting on certain molecules released from exosomes, promoting exosome release may be a protective method against drug stress conditions to eliminate cellular damage.

Ticagrelor is a purinergic drug, it has been widely used in patients with acute coronary syndrome (ACS) and myocardial infarction (176). Existing studies have reported that ticagrelor enhanced the release of cell-derived exosomes from the anti-hypoxic cardiac group by increasing cell proliferation *in vitro* (177). In addition, extracellular vesicles derived from cardiomyocytes pretreated with ticagrelor have a protective effect on hyperglycemic cardiomyocytes by attenuating oxidative and endoplasmic reticulum stress (178). Recently, Kulshreshtha et al. confirmed that simvastatin, a HMG CoA inhibitor, mediates exosome release by altering MVBs transport and that its mediated reduction in monocyte-derived exosome secretion is protective *in vitro* model of atherosclerosis (179). Likewise, exosomes derived from mesenchymal stem cells (MSCs) pretreated with atorvastatin (ATV) dramatically enhanced the efficacy of treatment of acute myocardial infarction (AMI), possibly by enhancing endothelial cell function through paracrine mechanisms (180). It was also found that extracellular vesicles of cannabis with high cannabidiol (CBD) content induce anticancer signaling in human hepatocellular carcinoma (181).

Notably, Zhang et al. reported that neutral sphingomyelinase inhibitor (Manumycin A) and ketoconazole had no effect on exosomes released from normal cells, but affected exosomes released from tumor cells, which is crucial for disease treatment (182). It remains to be further investigated whether this can be mediated by the influence of proto-oncogenes and/or oncogenes in the tumor cells or by other factors. Considering that most of the experiments were performed on tumors, it remains to be further explored how these drugs affect the cancer phenotype by influencing the exosome release process and thus the cancer phenotype. Furthermore, we need to be aware that drugs have certain side effects. In the future, there is also need to focus on what doses of these drugs should be used to reach specific sites of cancer in a particular way to inhibit or promote exosome release as a form of cancer treatment.

In addition, other factors such as food compounds (183, 184), temperature (185, 186), radiotherapy (187) and chemotherapy (188, 189)affect intercellular communication mechanisms by mediating exosome release process, which allows exosomes to perform different functions and then contributes to the diagnosis and treatment of diseases.

## Exosome molecular cargoes are used as disease diagnostic biomarkers

Exosome components indicate the biological state of the initiating cells and reflect the health status of the organs. Recently, more and more studies have shown that EVs contents can be applied in the diagnosis of various diseases (13, 190–193). This section summarizes the biomarkers that may become clinically common diseases in several major classes of molecular cargoes.

### Exosomal nucleic acids

#### Exosomal mRNAs

Messenger RNA (mRNA) is a single-stranded ribonucleic acid that carries genetic information and can guide protein synthesis. mRNA is not only an important exosome cargo, but also acts as a functional modulator in cancer cell-derived exosome processes (194). In order to study the diagnostic performance of circulating exosomal messenger RNA (emRNA) and tissue mRNA in prostate cancer (PCa) patients, Ji et al. (195) demonstrated circulating emRNA is more advantageous as a diagnostic biomarker in PCa patients. Recipient operating characteristic curve (ROC) analysis indicated that the AUC value of circulating emRNA in PCa screening and diagnosis was 0.948 and 0.851 respectively. Furthermore, the six molecules in emRNA including CDC42, IL32, MAX, NCF2, PDGFA and SRSF2 were upregulated in the screening and diagnosis of PCa patients compared to healthy controls (195). Similarly, Shephard et al. (88) said that serum-derived EV-mRNA has great potential for the differential diagnosis of prostate cancer. Among these, increased serum-derived EV-mRNA CTGF molecule or decreased EV-mRNA CAV1 molecule were closely associated with the rate of disease progression, and the AUC values of CTGF and CAV1 were 0.8600 and 0.8100 respectively. However, serum PSA could not predict disease progression, suggesting that EV-mRNA CTGF and CAV1 are superior to PSA in predicting disease progression (88). Another study proved that mRNA index of membrane matrix type 1 metalloproteinase (MT1-MMP) was significantly up-regulated in gastric cancer (GC) patients, with an AUC of 0.788, sensitivity of 63.9% and specificity of 87.1%, while the AUC value of serum CEA was only 0.655. Meanwhile, the combined exosomes diagnosis of mRNA(MT1-MMP) and CEA (AUC=0.821) was significantly better than the detection of mRNA (MT1-MMP) or CEA separately in identifying GC patients. In addition, it has been shown that exosomal epithelial growth factor receptor (EGFR) mRNA may be a potential predictor of glioblastoma (196). Serum exosome mRNA(MT1-MMP) was significantly associated with tumor differentiation, depth of invasion, lymphatic metastasis, distal metastasis and TNM stage (89). In brief, these studies show that exosomal mRNAs may have the potential to act as cancer biomarkers, but their specificity for the disease should be further investigated.

#### Exosomal miRNAs

MiRNA is a class of small endogenous noncoding RNA composed of 18-24 nucleotides, and the miRNA that delivered to the recipient cells can regulate various gene expression by preventing translation and inducing mRNA degradation (197). In addition, Exosomal miRNAs are more stable than free miRNAs as they are protected from degradation owing to RNase activity in biofluids (198). Recent studies have revealed that exosomal miRNAs may serve as potential biomarkers in certain cancers. For example, Yang et al. (104) found that exosomal miR-423-5p level was highly expressed in gastric cancer (GC) patients serum, and the AUC values of exosomal miR-423-5p, serum CEA and CA-199 were 0.763, 0.596 and 0.607 respectively (104). Notably, the combined detection of miRNAs can improve diagnostic accuracy. Huang et al. (199) found that six miRNAs were significantly higher expressed in serum exosomes of GC patients, whose AUC values were 0.627 (miR-10b-5p), 0.652 (miR-132-3p), 0.637 (miR-185-5p), 0.683 (miR-195-5p), 0.637 (miR-20a-3p) and 0.652 (miR-296-5p). At the same time, the AUC of the combined detection of the six miRNAs was 0.703, significantly improved the diagnostic accuracy of GC patients (199). Another study showed that the AUC values of serum exosomal miR-19b-3p and miR-106a-5p were 0.813 and 0.806 respectively. The AUC of their combined diagnosis was 0.826 (154). Similarly, in urinary exosomes from patients with renal clear cell carcinoma (ccRCC), different combinations of miRNAs, including miR-126-3p + miR-449a, miR-126-3p + miR-34b-5p, miR-126-3p + miR-486-5p, miR-25-3p + miR-34b-5p, miR-34b-5p, miR-2 b-5p-34 b-5p and miR-150-5 p + miR-126-3p have been reported to be potential diagnostic biomarkers in ccRCC patients. The sensitivities of these six combinations were 60.6%, 67.3%, 52.9%, 73.1%, 74%, and 61.5% respectively. Accordingly, specificities were 100%, 82.8%, 95.8%, 79.3%, 72.4%, and 82.8%, respectively. Furthermore, the targets of these miRNAs may be related to cell cycle regulation, tumorigenesis and angiogenesis (200). Muramatsu-Maekawa et al. (201) stated that miRNA-4525 in serum EVs is significantly higher expression in patients with advanced renal cell carcinoma (RCC) (201). Initially, serum exosomal miR-17-5p and miR-21 levels were considered as potential biomarkers for the differentiation of primary adenocarcinoma (PC). The mean levels of miR-17-5p and miR-21 were significantly higher in PC patients than in healthy controls (HPs) and non-PC groups, and the AUC values for miR-17-5p and miR-21 were 0.887 and 0.897 respectively, and the sensitivity and specificity of miR-17-5p were 72.7% and 92.6%, and 95.5% and 81.5% for miR-21 respectively (93). Subsequently, serum exosomal miRNAs (including miR-1246, miR-4644, miR-3976, and miR-4306) were also proposed as potential diagnostic biomarkers for pancreatic cancer (202). Notably, Manterola et al. (203) found that serum exosomal miR-320 and miR-574-3p were significantly higher expression in patients with glioblastoma multiforme (GBM) as compared with healthy controls, and ROC curve analysis indicated AUC for exosomal miR-320 and miR-574-3p of 0.720 and 0.738 respectively (203). In conclusion, exosomal miRNAs may be regarded as potential biomarkers of diseases.

#### Exosomal lncRNAs

In addition to miRNAs, exosomal lncRNAs are also attractive as potential diagnostic biomarkers. Long noncoding RNA (lncRNA) exists in the nucleus or cytoplasm, and they can interact with DNA, RNA, or proteins (204). Several studies have shown that exosomal lncRNAs may have the potential to act as biomarkers for cancer diagnosis. For example, plasma expression of lncUEGC1 was significantly higher in gastric cancer (GC) patients of stage I or II, and plasma exosomal lncUEGC1 (AUC =0.8760) was significantly superior to serum CEA (AUC = 0.6614). This suggests that exosomal lncUEGC1 may be a highly potential sensitive biomarker in early gastric cancer diagnosis (107). In addition, serum exosomal lncRNA HOTTIP was found to be a potential diagnostic index for gastric cancer patients. The ROC curve indicated that HOTTIP had high diagnostic value with an AUC value of 0.827 and higher diagnostic power than CEA, CA19-9 and CA72-4 (AUC values of 0.653, 0.685 and 0.639, respectively). It’s important that HOTTIP expression level was significantly correlated with the depth of invasion and TNM stage in gastric cancer (108). Another study confirmed that circulating exosomal long noncoding RNA-GC1 (lncRNA-GC1) expression could distinguish early gastric cancer patients and healthy controls, and ROC curve indicated that better exosomal lncRNA-GC1 (AUC=0.9033) compared to serum CEA, CA72-4 and CA19-9 (AUC values of 0.5987,0.6816 and 0.6482, respectively) (109). In addition, LINC00152 was also significantly elevated in the plasma exosomes of gastric cancer patients. Elevated exosomal LINC00152 was considered as a potential diagnostic indicator of gastric cancer with an AUC value of 0.657 (205). Similarly, Xiao et al. (110) demonstrated that lncRNA CCAT1 was significantly higher in serum EVs in gastric cancer patients than in healthy controls, chronic gastritis or dysplasia, with EVs lncRNA CCAT1 having an AUC of 0.890, sensitivity of 79.6%, specificity of 92.6%, while EVs lncRNA CCAT1 and embryo antibody combinations of 0.910 of 80.5% and 92.6% respectively. Moreover, EVs lncRNA CCAT1 may promote gastric cancer cells proliferation, migration and invasion through c-Myc or Bmi-1 upmodulation (110).

#### Exosomal circRNAs

Circular RNA (circRNA) is a class of noncoding RNA, mainly produced by pre-mRNA splicing. In contrast to miRNA, circRNA is abnormally stable, conserved and has cells or tissue-specific expression pattern (206). Exosomal circRNAs are anti-degradative, and its secretion into the extracellular environment can be used for many biological applications. Importantly, exosomal circRNAs may serve as novel diagnostic biomarkers. For example, Shao et al. (115) found that the expression of plasma exosomal hsa_circ_0065149 was significantly reduced in gastric cancer patients compared with healthy cohort, suggesting that reduced hsa_circ_0065149 is a potential diagnostic biomarker for gastric cancer (AUC=0.640) (115). Similarly, Xie et al. (116) found significant higher serum circSHKBP1 level in gastric cancer patients with a sharp decrease in exosomal circSHKBP1 after surgical resection of the tumor (116). A previous study in plasma EVs from breast cancer patients proved that nine circRNAs (including hsa_circ_0002190, hsa_circ_0007177, hsa_circ_0000642, hsa_circ_0001439, hsa_circ_0001417, hsa_circ_0005552, hsa_circ_0001073, hsa_circ_0000267 and hsa_circ_04004) combinations display maximum AUC values, and the AUC is 0.83 (207). In cholangiocarcinoma, circ-0000284 was significantly elevated in cholangiocarcinoma cell lines, its tissues and plasma exosomes, and higher expression of circ-0000284 promoted the migration, invasion and proliferation capacity of cholangiocarcinoma cells *in vitro* and *in vivo* (208). Therefore, the exosomal circ-0000284 could be used as a potential metastatic diagnostic biomarker. Circulating exosomal hsa-circ-0004771 was significantly upregulated in colorectal cancer (CRC) patients and AUC values of hsa-circ-0004771 were 0.59, 0.86 and 0.88 in differentiating between intercancer, stage I/II and CRC patients and healthy controls respectively, suggesting that hsa-circ-0004771 could serve as a new potential diagnostic biomarker for CRC patients (209). Moreover, exosomal circRNAs in serum and urine have the potential to act as diagnostic biomarker for idiopathic membranous nephropathy (IMN) (210). In short, these studies suggest that exosomal circRNAs have the possibility of act as biomarkers for disease diagnosis. However, whether its expression levels are specific for different disease and tumor subtypes remains to be further investigated.

### Exosomal proteins

In addition to nucleic acids, exosomal proteins have been found to act as potential biomarkers for diseases. Because exosomes contain multiple protein molecules that reflect the characteristics of its parental cells (211). Exosomal proteins have been found in different body fluids (including serum, plasma, urine, saliva and cerebrospinal fluid) and may have the potential to serve as biomarkers for cancer diagnosis. For example, the cell surface proteoglycan Glypican-1 (GPC1), a member of the heparan sulfate proteoglycan family, is a widespread cell surface protein (212). It has been suggested that GPC1-positive exosomal was highly expressed in the serum of pancreatic cancer patients, and the diagnostic power of the exosomal protein GPC1 (AUC = 1.0) was significantly better than CA19-9 (AUC =0.739) in distinguishing pancreatic cancer patients from healthy controls. CA19-9 serum levels cannot distinguish patients with intraductal papillary mucinous tumors (PCPL) from healthy controls, while GPC1-positive serum exosomal had 100% sensitivity and specificity in all stages of pancreatic cancer (e. g.: cancer in situ, stage I, and stage II-IV) (120). Similarly, the exosomal protein GPC1 expression was significantly increased in both plasma and tissue samples of colorectal cancer (CRC) patients, and both normalized after surgical treatment (213). Another study indicated that the downregulation of serum exosomal Gastrokine 1 (GKN1) protein may be a valid diagnostic biomarker in gastric cancer patients (129).

Recently, the proteomic analysis of extracellular vesicles and granules (EVP) from 426 human samples derived from tissue explants (TE), plasma and other body fluids by Hoshino et al. (214). They confirmed that CD63 and flotillins were heterogeneous in plasma and tissue EVP. And Leucine-rich repeat protein 26 (LRRC26), ATP-dependent translocase ABCB1 (ABCB1), Bile salt export pump (ABCB11), Adhesion G protein-coupled receptor G6 (ADGRG6), Desmosomes-1 (DSC1), Desmoglein-1 (DSG1), Keratin and Plasminogen-like protein B (PLGLB1) were present only in plasma-derived EVP in patients with pancreatic cancer (PaCa), absent or extremely low expression in tumor tissue (TT) and adjacent normal tissue (AT) -derived EVP. This suggests that these proteins have the potential to act as characteristic tumor-associated EVP proteins. In addition, they said that EVP proteins can distinguish between cancer in the early stages of pancreatic cancer (PaCa) and lung adenocarcinoma (Luca) patients (214). It is interesting that, by proteomic analysis of Sun et al. (215), Annexin family members (Annexin A1, A2, A3, A5, A6, A11), Nitrogen permease regulator 2-like protein(NPRL2), Carcinoembryonic antigen-related cell adhesion molecule 1(CEACAM1), Mucin 1(MUC1), Prominin-1 (PROM1), Histone H4 (HIST1H4A) and Tumor necrosis factor alpha-induced protein 3 (TNFAIP3) were associated with lung cancer, which is helpful in lung cancer diagnosis (215). The expression levels of plasma exosomal Tim-3 and Galectin-9 protein molecules were significantly increased in non-small-cell lung cancer (NSCLC) patients, as compared with healthy controls. It’s important that exosomal Tim-3 and Galectin-9 expression levels were positively correlated with clinicopathological features such as patient age, tumor size, distant metastasis and cancer stage. Moreover, exosomal Tim-3 is also associated with lymph node metastasis. Therefore, exosomal Tim-3 and Galectin-9 may serve as potential biomarkers for the clinical application of NSCLC (216). All of these findings suggest that exosomal proteins have the potential to serve as biomarkers for disease diagnosis. In the future, we still need to focus on the expression levels of specific proteins in a certain disease.

### Exosomal lipids

Lipid molecules in exosomes are mainly used to maintain their external morphology. It has been reported that lipid molecules in EVs can not only protect nucleic acids and protein contents from harmful stimuli in the extracellular environment, but also exert bioactive functions to participate in tumor biological processes as signaling molecules (217, 218). It has been shown that lipid molecules in exosomes can also be used as potential biomarkers in cancer patients (136, 219–222). Among them, the expression levels of phosphatidylcholine(PC), phosphatidylethanolamine (PE),phosphatidylinositol(PI),sphingomyelin(SM),ceramide(Cer) and cholesterol are various in difference diseases (150, 223–225).

Previously, Skotland et al. (223) pointed out that urinary exosomal lipid molecules (such as phosphoresterdylserine and lactoceramide) have potential as biomarkers in prostate cancer (134). Subsequently, Brzozowski et al. (226) performed lipid analysis in exosomes released from non-tumorigenic (RWPE1), tumorigenic (NB26) and metastatic (PC-3) prostate cell lines, and they found significant differences in lipid species abundance in cells of these three different prostate species. The abundance of Diacylglycerol (DG) and Triacylglycerol (TG) species were reduced in both the NB26 and PC-3 cell lines EVs as compared to the EVs in the RWPE1 cell line. However, in contrast to EVs in the RWPE1 cell line, EVs in the NB2 and PC-3 cell lines were rich in glycerophospholipids, while Cer and SM species do not differ much among the three cell lines (226). In addition, Exosomal lipid components have been detected in Hepatocellular Carcinoma (HepG2/C3a and Huh7 cells) (227), Melanoma (B16-F10 cells) (228), Glioblastoma (U87 cells) (229)and Pancreatic cancer (AsPC-1 cells) (230). Recently, Glover et al. (135) stated that the content of exosomal lipid molecules such as glycerophospholipids, glycerolips, and sterols is reduced in the urine of patients with hereditary-trypsinaemia (135). Overexpression of exosomal lipid molecules such as acid sphingolipase in the cerebrospinal fluid of multiple sclerosis(MS) patients is strongly associated with disease severity, creating new opportunities for the diagnosis and treatment of the disease (137). Furthermore, sphingomyelin, derived from EVs in tumor cells, promotes endothelial cell migration and angiogenesis during tumor growth and metastasis (231). To sum up, this suggests that the great potential of EVs lipid molecules for cancer diagnostic biomarkers.

### Summarizing the role of exosomal molecular cargoes in cancer diagnosis

In conclusion, exosomal nucleic acids, proteins and lipid molecular cargoes in different body fluids have broad application prospects as cancer diagnostic biomarkers (**Table 3**). Previous researches have shown that exosomal molecular cargoes are differentially expressed in body fluids, and exosomal molecular cargoes with higher AUC values may effectively distinguish cancer patients from healthy individuals (232–234). It is worth noting that the combined detection of multiple potential exosome molecular cargoes may provide a rapid, reliable and non-invasive aid to the diagnosis of diseases. In addition, that exosomes used as diagnostic biomarkers also requires consideration of all preanalytical variables associated with sample collection, such as whole blood (or other biofluid) treatment, hemolysis interference, and other contaminant interference (235). In the future, we should also focus on large-scale preparation and standardized protocols for exosomes analysis, and need advanced techniques to minimize contaminants in the samples as well.

**Table 3:** Exosomal molecular cargoes are used as biomarkers for disease diagnosis.

Potential Molecular Cargoes	Expression	Diseases	Source	Isolation	AUC	Clinical Significance	References
***mRNAs***							
CTGF	↑	Prostate cancer	Serum	UC	0.8600	Early diagnosis & Prognostic monitoring	(88)
CAV1	↓	Prostate cancer	Serum	UC	0.8100	Early diagnosis & Prognostic monitoring	(88)
THBS1	↓	Prostate cancer	Serum	UC	0.8200	Early diagnosis	(88)
TIMP2	↓	Prostate cancer	Serum	UC	0.8000	Early diagnosis	(88)
MT1-MMP	↑	Gastric cancer	Serum	CRG	0.7880	Diagnosis, Treatment, and Prognosis	(89)
hnRNPH1	↑	Hepatocellular carcinoma	Serum	CRG	0.8650	Early diagnosis & Prognostic monitoring	(90)
***miRNAs***							
miR-141	↑	Prostate cancer	Serum	PC	0.8694	Early diagnosis	(91)
miR-196a-5p	↓	Prostate cancer	Urine	UC	0.7300	Early diagnosis	(92)
miR-501-3p	↓	Prostate cancer	Urine	UC	0.6900	Early diagnosis	(92)
miR-196a	↓	Prostate cancer	Urine	UC	0.9200	Early diagnosis	(92)
miR-17-5p	↑	Pancreatic cancer	Serum	UC	0.8870	Early diagnosis & Prognostic monitoring	(93)
miR-196a	↑	Pancreatic cancer	Plasma	UC	0.8100	Early diagnosis & Prognostic monitoring	(94)
miR-1246	↑	Pancreatic cancer	Saliva	CRG	0.8140	Early diagnosis	(95)
miR-4644	↑	Pancreatic cancer	Saliva	CRG	0.7630	Early diagnosis	(95)
miR-101	↓	Ovarian cancer	Serum	PC	—	Early diagnosis & Treatment assessment	(96)
miR-224	↑	Hepatocellular carcinoma	Serum	PC	0.9100	Early diagnosis & Prognostic monitoring	(97)
miR-92b	↑	Hepatocellular carcinoma	Serum	PC	0.9250	Early diagnosis of recurrence after living donor liver transplantation (LD LT)	(98)
miR-122	↑	Hepatocellular carcinoma	Serum	PC	0.9900	Early diagnosis	(99)
miR-92b	↑	Colorectal cancer	Plasma	UC	0.7930	Early diagnosis	(100)
miR-122	↑	Colorectal cancer	Serum	PC	0.8900	Early diagnosis & Prognostic monitoring	(101)
miR-520c-3p	↑	Nonsmall-cell lung cancer	Serum	UC 、PC	0.8190	Early diagnosis	(102)
miR-1274b	↑	Nonsmall-cell lung cancer	Serum	UC 、PC	0.7880	Early diagnosis	(102)
miR-15a-5p	↑	Endometrial carcinoma	Plasma	PC	0.8130	Early diagnosis	(103)
miR-423-5p	↑	Gastric cancer	Serum	PC	0.7630	Early diagnosis & Prognostic monitoring	(104)
miR-15b-3p	↑	Gastric cancer	Serum	UC	0.8200	Early diagnosis & Prognostic monitoring	(105)
miR-4732-5p	↑	Epithelial Ovarian cancer	Plasma	CRG	0.8890	Early diagnosis	(106)
***lncRNAs***							
lncRNA-UEGC1	↑	Gastric cancer	Plasma	UC	0.8760	Early diagnosis	(107)
lncRNA-HOTTIP	↑	Gastric cancer	Serum	UC	0.8270	Early diagnosis & Prognostic monitoring	(108)
lncRNA-GC1	↑	Gastric cancer	Serum	UC	0.9033	Early diagnosis	(109)
lncRNA-CCAT 1	↑	Gastric cancer	Serum	UC, CRG	0.8900	Early diagnosis	(110)
lncRNA-UCA1	↑	Bladder cancer	Serum	CRG	0.7530	Early diagnosis	(111)
lncRNA - PTENP1	↓	Bladder cancer	Plasma	CRG	0.7430	Early diagnosis & Prognostic monitoring	(112)
lncRNA - TERC	↑	Bladder cancer	Urine	UC	0.8360	Early diagnosis & Prognostic monitoring	(112)
lncRNA -LINC00635	↑	Hepatocellular carcinoma	Serum	CRG	0.7500	Early diagnosis & Prognostic monitoring	(113)
lncRNA -HOTAIR	↑	Glioblastoma	Serum	CRG	0.9130	Early diagnosis & Prognostic monitoring	(114)
***circRNAs***							
hsa_circ_0065149	↓	Gastric cancer	Plasma	CRG	0.6400	Early diagnosis & Prognostic monitoring	(115)
circSHKBP1	↑	Gastric cancer	Serum	PC	—	Early diagnosis & Prognostic monitoring	(116)
circ-KIAA1244	↓	Gastric cancer	Plasma	CRG	0.7481	Early diagnosis	(117)
circSATB2	↑	Lung cancer	Serum	UC	0.6600	Early diagnosis	(118)
circLPAR1	↓	Colorectal cancer	Plasma	CRG	0.8580	Early diagnosis	(119)
***Proteins***							
glypican-1	↑	Pancreatic cancer	Serum	UC	1.0000	Early diagnosis	(120)
Survivin	↑	Prostate cancer	Plasma	UC	—	Early diagnosis & Prognostic monitoring	(121)
EphrinA2	↑	Prostate cancer	Serum	UC	0.7666	Early diagnosis	(122)
MAGE 3/6	↑	Ovarian cancer	Plasma	UC	—	Early diagnosis & Treatment assessment	(123)
Epcam-CD63	↑	Colorectal cancer	Plasma	UC	0.9600	Early diagnosis & Prognostic monitoring	(124)
TRIM3	↓	Gastric cancer	Serum	PC	—	Early diagnosis	(125)
MUC1	↑	Nonsmall-cell lung cancer	Plasma	CRG	0.6850	Early diagnosis	(126)
Del-1	↑	Breast cancer	Plasma	ELISA(CD63* capture)	0.9610	Early diagnosis	(127)
Fibronectin	↑	Breast cancer	Plasma	ELISA(CD63* capture)	0.7700	Early diagnosis	(128)
GKN1	↓	Gastric cancer	Serum	UC	1.0000	Early diagnosis & Treatment assessment	(129)
CP	↑	Renal cell carcinoma	Urine	UC	1.0000	Early diagnosis	(130)
PODXL	↑	Renal cell carcinoma	Urine	UC	1.0000	Early diagnosis	(130)
EpCAM	↑	Metastatic breast cancer	Plasma	UC	0.9709	Early diagnosis	(131)
PD-L1	↑	Nonsmall-cell lung cancer	Serum	UC	0.9700	Early diagnosis	(132)
CD24	↑	Ovarian cancer	Plasma	UC	1.0000	Early diagnosis	(133)
EpCAM	↑	Ovarian cancer	Plasma	UC	1.0000	Early diagnosis	(133)
FRα	↓	Ovarian cancer	Plasma	UC	0.9950	Early diagnosis	(133)
***Lipids***							
Phosphatidylserine (PS) 18:1/18:1 and lactose ceramide (d18:1/16:0)	↑	Prostate cancer	Urine	UC	0.9890 (In combination)	Early diagnosis	(134)
Glycerophospholipids, glycerolips and sterols	↓	Hereditary alpha-tryptophanemia	Urine	UC	–	Early diagnosis	(135)
PC (P-14:0/22:2)	↑	Pancreatic cancer	Serum	PC	–	Early diagnosis & Prognostic monitoring	(136)
Acid sphingomyelinase	↑	Multiple sclerosis	Cerebrospinal fluid	UC	0.7700	Early diagnosis & Treatment assessment	(137)

↑, increased; ↓, decreased; –, unrevealed; UC, ultracentrifugation; PC, precipitation; CRG, Commercial reagents.

## Conclusion

In this review, we illustrate that exosomal molecular cargoes participate in exosome biogenesis, which is a complex process that may vary in cargoes or cellular origin. In addition, the regulation of exosome biogenesis processes involves the coordination of many different molecular cargoes and signaling mechanisms, mainly dominated by ESCRT-dependent, lipid raft and tetraspanin protein mechanisms, and Rab proteins further assists cargo sorting and exosome release. Notably, this cargo molecules interact with each other to mainly mediate exosome biogenesis by regulating the negative curvature of the cell membrane (236). So far, ESCRT and ceramide pathways are established for exosome biogenesis.

Furthermore, exosomes and their molecular cargoes are elaborated as effective tools for the diagnosis of cancer. Although tissue biopsy is still the gold standard for tumor diagnosis, but it is invasive. An ideal diagnostic approach for cancer should accurately detect tumor-specific biomarkers using non-invasive techniques at the pre-metastatic stage (237). Most of the current molecules used as tumor diagnostic biomarkers are based on detecting the higher expression molecules above the threshold in healthy individuals. For instance, PSA and CEA serve as diagnostic biomarkers for prostate cancer and gastrointestinal cancer respectively, and these biomarkers are significantly elevated only at tumor progression state (238). Since exosomes are present in most body fluids and their stability properties, and the molecular cargoes carried by exosomes reflects the genetic or signaling changes in the cancer cells of origin. If it would be detected earlier as biomarkers, so as to achieve a means of treating the disease, it would make exosomes potentially replace invasive biopsies as cancer diagnostic biomarkers of important clinical significance (239, 240).

Understanding the process of exosome biogenesis is an important part of the research and physiological significance of exosomes function, especially for disease diagnosis, treatment, and prognosis. Controlling exosome generation in pathological states may serve as a therapeutic opportunity to reduce tumorigenesis. However, it is still challenging to investigate the whole mechanism of exosome biogenesis. Because the exosome formation pathway may be different according to different cell types, some specific molecules will participate in multiple processes, leading to the exact mechanism of action of many molecules is not clear, for which their heterogeneity may be a disadvantage of their use as biomarkers. It is worth noting that most studies in the field of exosomes are conducted *in vitro*, and the laboratory culture conditions or technical methods also affect the biological characteristics of exosomes (241). Therefore, special attention should also be paid to the methods of exosomes extraction used in each study. How to promote the yield and purity of exosomes is a top priority, which has been a bottleneck limiting their translational applications. Recent studies have shown that appropriate combinations of several methods for extracting and purifying exosomes can effectively improve the above problems, and how to integrate them for optimum results remains to be further investigated. More work needs to be done in the future to elucidate the role of exosomes in diseases progression, with particular attention to the precise mechanisms by which exosome biogenesis pathways influence cellular function. The questions will be raised such as, will different biogenesis pathways produce vesicles with different or similar functions? Will there be any correlation between vesicles produced by this biogenesis pathways? This will be useful for treatments involving the pathological mechanisms of exosomes. Understanding the physiological effects and how they can be induced into pathological factors is crucial when developing new therapeutic strategies.

## Author contributions

XY searched for literature and wrote the first draft of this article,JC revised the manuscript and developed the main content of this manuscript. SF provided great help for polishing the manuscript. DH, TY, ZL, XW, MZ, and JW were involved in edited the manuscript. TZ supervised the project and contributed to the revision of the final manuscript. All authors contributed to the article and approved the submitted version.

## Funding

This work was supported by the Key R&D Planning Project of Jiangxi Science and Technology Commission, China (No. 20203BBGL73126).

## Acknowledgments

We thank Yaojiang Que edited the figure, Zhigang Li reviewed the manuscript and polished the grammar.

## Conflict of interest

The authors declare that the research was conducted in the absence of any commercial or financial relationships that could be construed as a potential conflict of interest.

## Publisher’s note

All claims expressed in this article are solely those of the authors and do not necessarily represent those of their affiliated organizations, or those of the publisher, the editors and the reviewers. Any product that may be evaluated in this article, or claim that may be made by its manufacturer, is not guaranteed or endorsed by the publisher.
